# Photothermal Antibacterial and Osteoinductive Polypyrrole@Cu Implants for Biological Tissue Replacement

**DOI:** 10.3390/ma17153882

**Published:** 2024-08-05

**Authors:** Tianyou Zhou, Zeyan Zhou, Yingbo Wang

**Affiliations:** 1 College of Control Engineering, Xinjiang Institute of Engineering, 1350 Aidinghu Road, Urumqi 830023, China; tyzhou_power@163.com; 2 College of Materials Science and Engineering, Hunan University, 2 South Lushan Road, Changsha 410082, China; zhouzeyan@hnu.edu.cn; 3 College of Chemical Engineering, Xinjiang Normal University, 102 Xinyi Road, Urumqi 830054, China

**Keywords:** multiple antibacterial mechanisms, potent-durable antibacterial, osteoinductive properties, photothermal, biomaterial coating

## Abstract

The treatment of bone defects caused by disease or accidents through the use of implants presents significant clinical challenges. After clinical implantation, these materials attract and accumulate bacteria and hinder the integration of the implant with bone tissue due to the lack of osteoinductive properties, both of which can cause postoperative infection and even lead to the eventual failure of the operation. This work successfully prepared a novel biomaterial coating with multiple antibacterial mechanisms for potent and durable and osteoinductive biological tissue replacement by pulsed PED (electrochemical deposition). By effectively regulating PPy (polypyrrole), the uniform composite coating achieved sound physiological stability. Furthermore, the photothermal analysis showcased exceptional potent photothermal antibacterial activity. The antibacterial assessments revealed a bacterial eradication rate of 100% for the PPy@Cu/PD composite coating following a 24 h incubation. Upon the introduction of NIR (near-infrared) irradiation, the combined effects of multiple antibacterial mechanisms led to bacterial reduction rates of 99% for *E. coli* and 98% for *S. aureus* after a 6 h incubation. Additionally, the successful promotion of osteoblast proliferation was confirmed through the application of the osteoinductive drug PD (pamidronate disodium) on the composite coating’s surface. Therefore, the antimicrobial Ti-based coatings with osteoinductive properties and potent and durable antibacterial properties could serve as ideal bone implants.

## 1. Introduction

With the continuous progress in medical technology and material science, and the wide application of implants in orthopedics, bone surgery, dentistry, etc., implants have become an important tool to promote human tissue repair. Ti (Titanium) and its alloys possess superior biocompatibility, biocorrosion resistance, and a low elastic modulus [1,2,3,4]. This enables them to provide the requisite mechanical strength and elasticity for successful replication of bone formation in orthopedic uses. However, infection problems associated with Ti substrates are common. Bacterial infections, usually caused by bacterial attachment and biofilm formation, can lead to high morbidity and even high mortality. Many different approaches have been taken to prevent implant-associated infections [5]. Modifications such as the use of ultraviolet light on the surface of the implant or the implementation of crystallization grafting can help reduce bacterial attachment. In recent years, more emphasis has been placed on developing implant surface coatings that can prevent bacterial infections and promote tissue repair or induce tissue regeneration. However, the adhesion of current coatings to the surface of the implant is not strong enough and is easily damaged, which affects its antibacterial effect [6]. Therefore, in order to solve this problem, various methods have been developed to graft antibiotics to Ti surfaces such as vancomycin or other antimicrobial peptides. These grafting methods improve the binding properties of the coating and the implant surface, but the antibacterial mechanism of the coating on the surface of the implant is single, making the antibacterial life of the antibacterial material limited, which affects its lasting antibacterial effect. Therefore, it is necessary to conduct further research and develop new antibacterial coating technologies that can simultaneously satisfy the persistence and synergistic effect of multiple antibacterial mechanisms.

At present, the antibacterial coatings composed of osteoinductive drugs prepared on the surface of Ti mainly contain antibiotics, antibacterial peptides, copper, etc. Zhang et al. [7] designed a Ti-SRP-Van (antibiotic) composite coating by self-assembly technology, and the bacteriostatic activity reached 66.7%, which was relatively low. Moreover, antibiotics application for a prolonged period would cause bacterial resistance. Abbasizadeh [8] combined electrospinning and self-assembly methods to construct a Ti/SF (silk fibroin)/HA (Hydroxyapatite)/antibacterial peptide composite coating with a bacteriostatic activity of 100%, but antibacterial peptides have high environmental requirements. Tabesh [9] constructed a chitosan/Cu composite coating on the surface of Ti via electrophoretic deposition. The introduction of Cu^2+^ endowed the composite coating with superior antibacterial properties, and the bacteriostatic activity against *S. aureus* (*Staphylococcus aureus*) and *E. coli* (*Escherichia coli*) reached 100% and 75%, respectively. However, high concentrations of metal ions can be toxic to cells. Precise regulation of metal ion content, concentration, and distribution on the coatings could reduce their cytotoxicity. Our group previously developed a PPy-regulated Cu-containing composite coating on the surface of Ti substrate via pulsed electrochemical deposition [10]. The Cu-NPs were uniformly distributed in the composite coating and displayed good physiological stability, revealing that the introduction of the modulator PPy regulated metal ions. Cu is an essential trace element in the human body, which can bind to the FGF-2 growth factor [11], promote the proliferation of vascular endothelial cells, stimulate angiogenesis, and induce MSCs (mesenchymal stem cells) to differentiate into osteoblasts [12,13]. Meanwhile, Cu has a broad antibacterial spectrum and can effectively prevent bacterial resistance [14]. Hence, constructing an osteoinductive Ti-based composite coating with precise modulation of Cu nanoparticles is an effective antibacterial strategy.

Studies have shown that the long-term application of antibacterial materials would induce the mutation and evolution of the bacteria, leading to secondary infection and failure of surgery [15]. Thus, shortening the antibacterial period is the key to the success of clinical surgery. It is reported that NIR light irradiation could elevate the local temperature of the materials, which increases the permeability of bacterial cell membranes [16]. At the same time, high temperature induces denaturation of bacterial proteins and causes bacterial death [17,18]. Hence, PTT (photothermal therapy) based on NIR light irradiation has been extensively used [19]. Cu-NPs are highly sensitive to NIR light and could generate ROS (reactive oxygen species) under the catalysis of NIR light. The interaction of ROS with bacteria causes oxidative damage to the bacteria membrane, destroys their integrity, and makes the bacteria more susceptible to heat [20]. Moreover, Cu-NPs convert the absorbed NIR light into heat, which can destroy the bacteria membrane to induce cytoplasmic leakage, further enhancing the bactericidal efficacy [21]. Tao et al. [22] incorporated Cu-NPs into a hydrogel, combined with 808 nm NIR radiation to generate ROS, and effectively produced local heat. After incubation with *E. coli* or *S. aureus* for 6 h, its bacteriostatic activity increased by 41.1% and 42.3% compared with the non-radiated hydrogel groups. Chen et al. [23] also combined Cu-NPs with hydrogel, and the results showed that the Cu-NPs embedded in the hydrogel exhibited excellent photothermal conversion efficiency. Furthermore, after being irradiated for 10 min, it was observed that the temperature in the Cu-NP-doped hydrogel increased significantly, and the bacteriostatic activity was increased by 79%, suggesting its efficient and fast antibacterial properties. It is worth noting that Cu^2+^ can be released from dead bacteria and repeat the above cycle to achieve a long-term antibacterial effect. Cu-NPs can also enhance bone tissue repair by stimulating bone formation and angiogenesis. Therefore, introducing NIR-responsive PTT is an essential strategy for achieving rapid and potent composite coating sterilization.

Extensive research has identified osteoinductive coatings on the surface of Ti as effective mitigation measures. The introduction of drugs such as raloxifene, bisphosphonates, and calcitonin effectively curbs osteoclast activity, reducing bone resorption and facilitating bone repair [24,25]. A specific clinical bisphosphonate drug, pamidronate disodium, shows wide-ranging success in treating excess bone resorption in various conditions [26]. Building on previous works, Mukherjee [27] developed a mucoadhesive pressed tablet formulation loaded with pamidronate for systemic administration, exhibiting remarkable cellular proliferation promotion. In a related study, Kajiwara [28] developed a self-assembled HA/PD composite coating on the surface of Ti, effectively inhibiting osteoclast production and fostering osteogenic functions. Results have shown their effectiveness, but neither of these intended biomaterials exhibit inherent antibacterial properties, leaving them susceptible to implant-related infections. To address the aforementioned issues, it is crucial to expeditiously develop potent, durable Ti-based Pd composite coatings that possess a range of antibacterial mechanisms and sustainable anti-infective qualities.

Guo et al. [29] designed a DA-Ag/PLL (polylysine)/SA (sodium alginate) composite coating on the surface of Ti using the layer-by-layer self-assembly method. Though this composite coating showed good antibacterial and osteoinductive properties, its binding effect with the substrate was weak and easily loosened after implantation in vivo. He et al. [30] established a HA/PDA-LL-37 (peptide LL-37) composite on a Ti surface by the micro-arc oxidation method. However, this method requires high-temperature and high-pressure conditions, which would cause HA decomposition. The above analysis shows that the bonding strength of the coating prepared by the self-assembly method is weak, and the high-temperature condition of the micro-arc oxidation technique will decompose the HA materials. PED can precisely control the content and morphology of coating substances by adjusting electrochemical parameters, and the method is simple and easy to implement [31]. When the potential of the metal ion electrode is high, the electrochemical reaction will be too fast and induce particle agglomeration. Thus, it is necessary to add a regulator to achieve precise modulation. The conductive polymer PPy could be used as a regulator [32]. During the electrochemical deposition process, the Py monomer would self-polymerize, and its amino group could coordinate with Cu^2+^ to disperse the Cu-NPs evenly by effective regulation. In addition, PPy holds good electrical responsiveness [33,34]. Therefore, after external electrical stimulation, the composite coating can generate a weak electric field, thereby providing electrical signals to activate the differentiation of osteoblasts and promote new bone formation. Zhou et al. [35] fabricated PPy@PDA/HA film on the surface of Ti using a layer-by-layer in situ assembly method. When the voltage reached 600 mV, electrical stimulation of cells enhanced cell proliferation and facilitated new bone formation. Thus, using the PED method to drive the conductive PPy to modulate Cu^2+^, build a composite coating with uniformly distributed Cu-NPs and osteoinductive drug, and perform PPy-conducted electrical stimulation is an effective strategy to promote new bone formation.

**Scheme 1:**
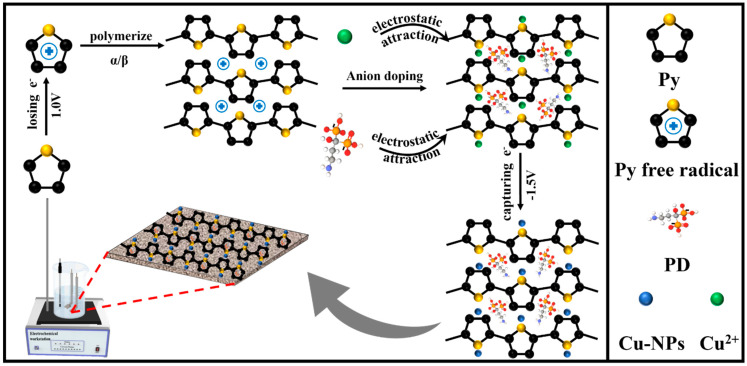
Mechanism diagram of constructing composite coating on Ti surface via the PED method.

In this study, PPy served as the regulator to uniformly disperse PD and Cu-NPs on the Ti surface via the PED method to engineer a PPy@Cu/PD composite coating, which has the NIR-responsive properties that can achieve potent, durable antibacterial effects and facilitate bone regeneration. First, Py (pyrrole) was oxidized to free radicals at a voltage of 1.0 V and then polymerized at the α or β position to generate PPy [36]. PPy was positively charged during the polymerization process and could be doped with anions. The negatively charged PD^2−^ was chelated into the main chain of PPy to neutralize its positive potential. Meanwhile, the pyrrole N atoms in the PPy were coordinated with Cu^2+^ [10], which was reduced to form Cu nanoparticles under the reduced voltage and evenly dispersed on the coating. The preparation mechanism is shown in Scheme 1. The biological activities, release profile of ion, antibacterial effect with/without NIR irradiation, osteoinduction, and other properties of this composite coating were characterized and analyzed.

## 2. Materials and Methods

### 2.1. Materials and Instruments

Titanium sheet (Baoji INT Medical Titanium Co., Ltd., Baoji, China), Py (98%, Sigma-Aldrich, St. Louis, MO, USA), PD (Kure Chemical Co., Ltd., Tokyo, Japan, purity~98%), and 1,3-Diphenylisobenzofuran (Shanghai Baishun Biotechnology Co., Ltd., Shanghai, China, AR). The two strains (*E. coli*: BeNa Culture Collection, Suzhou, China) and *S. aureus*: 25923 ATCC) were first verified in accordance with the conventional microbiological testing methods published by China’s National Food Safety Standards GB4789.3-2016 and GB4789.10-2016.

The crystal phase of the composite coating was detected using an XRD (X-ray powder diffractometer, D2-PHASER, 40 kV, BRUKER, Bremen, Germany). The TF-XRD (thin-film X-ray diffraction) was analyzed using a Cu-Kα (λ = 1.5418) X-ray source with the scanning rate at 0.5°·s^−1^ and a scanning range of 20–60°. The changes in the chemical structures of the oatings were detected by FTIR (Fourier transform infrared spectrometer, TERSOR27, BRUKER, Germany) scanning in the mid-infrared area from 4000 to 400 cm^−1^. The morphology and element content and distribution were determined by an SEM (scanning electron microscope, SU8010, HITACHI, Tokyo, Japan, 5.0 kV acceleration voltage) with EDS (Energy Dispersive Spectrometer). An electrochemical workstation (Shanghai Chenhua Instruments Co., Ltd., Shanghai, China, CHI660) was used to prepare samples. An ICP emission spectrometer (Inductively Coupled Plasma–Optical, ICAP6300, Thermo Elemental Corporation, Waltham, MA, USA) was used to quantify the amount of ion release. Physiological stability assays were conducted in a constant-temperature incubator (PBH-9082, Yiheng, China).

### 2.2. Preparation of Composite Coatings

Pretreatment of medical Ti sheets: The Ti sheets were polished with sandpaper until there were no black stains on the surface, exhibiting a silvery-white metallic luster. The polished titanium plates were then cleaned and polished under ultrasound with acetone and distilled water 3 times for 10 min each. Concentrated sulfuric acid, concentrated hydrochloric acid, and water (1:1:1, *v*/*v*/*v*) were mixed to prepare an acid solution. Then, Ti sheets were placed in it, sealed, and incubated at 60 °C for 6 h. After acid corrosion, the titanium sheets were cleaned with ultrasonic waves in distilled water for 10 min, and processed a total of 3 times. Next, Ti sheets were put in the 5 M NaOH solution, sealed, and reacted at 60 °C for another 1 h, followed by another ultrasonic cleaning with distilled water for 10 min, which was repeated three times. Finally, the pre-treated Ti sheets were dried for further experiments.

A uniform Py solution was obtained by completely dissolving 1 mL of Py in 1 L of deionized water under mechanical stirring. Then, 241.6 mg of Cu (NO_3_)_2_ was added to the Py solution to completely dissolve under mechanical stirring to obtain uniform and stable Py/Cu (NO_3_)_2_. Finally, it was supplemented with 1 × 10^−3^ g PD and stirred until well mixed. A three-electrode system, including the working electrode (Ti sheets), auxiliary electrode (Ti foil), and reference electrode (saturated calomel electrode), was placed in the electrolyte, and the sample was synthesized on the Ti sheets via controlled electrodeposition. The experiment was performed at an oxidative pulse width of 150 s and reductive pulse width of 450 s for 1 h at room temperature. After the reaction, the samples were left to dry naturally.

### 2.3. Investigation of Physiological Stability of Composite Coatings

The electrochemical corrosion experiment was performed in Ringer’s solution (900 mg·L^−1^ NaCl, 430 mg·L^−1^ KCl, 240 mg·L^−1^ CaCl_2_, 200 mg·L^−1^ NaHCO_3_) at 37 °C using a CHI660E (Shanghai Chenhua Instrument Co., Ltd., Shanghai, China) electrochemical workstation. The sample, platinum sheet, and saturated calomel electrode served as working electrodes, auxiliary electrodes, and reference electrodes, respectively. First, the samples were immersed in Ringer’s solution for 30 min to evaluate the open-circuit potential before a corrosion test. The voltage and scanning rate parameters were set to 0.25~−0.25 V and 0.5 mV·s^−1^, respectively, and dynamic scanning potentials were recorded. Epoxy was used to seal the sides of every sample to only expose 1 cm^2^ of surface area to the Ringer solution. Epoxy was used to cover the sides of each sample only. A tangent method was used to measure the current density (I_corr_) and corrosion potential (E_corr_) on the potential dynamic polarization curve. The cathodic and anodic polarization form tangents and their intersection shown as I_corr_ and E_corr_ represented the intersection of the tangents, of which values could be derived by Tafel extrapolation. Three measurements for every sample were recorded to assure a reliable comparison among curves.

### 2.4. Ion Release Profile Evaluation of Composite Coatings

The Cu/PD and PPy@Cu/PD coatings were placed in 40 mL PBS (Table S1) at a constant temperature of 37 °C, and PBS buffer was harvested and replaced on days 0.5, 1, 3, 5, 7, 10, and 14. The amount of released Cu^2+^ was quantified by ICP to study the physiological stability of composite coatings.

### 2.5. Evaluation of Drug Release Property of Composite Coatings

Different coatings were placed in 4 mL PBS solution at 37 °C from day 0.5 to day 30, and the release profile of PD^2−^ was measured (*n* = 3). The PBS solution was changed at seven time points, including days 0, 1, 3, 5, 7, 10, and 14, to collect the released solution. The concentration of PD^2−^ was detected and compared to the standard curve using a UV spectrophotometer (UV-2600, Shimadzu, Shanghai, China), and the drug release profile of the composite coating was calculated.

### 2.6. Photothermal Heating Assessment of Composite Coatings

The samples (*n* = 3) were placed in a 24-well plate, followed by adding 1 mL PBS. The temperature was recorded using a temperature probe. The initial temperature was recorded. Then, the infrared laser was fixed at a vertical height of 2–3 cm from the sample surface; adjusted to different irradiation powers, including 0.5, 1, 1.5, 2 W·cm^−2^; the temperature was recorded every 30 s for 10 min with a stopwatch; and the heating curve was plotted.

### 2.7. Photothermal Conversion Experiment

The sample preparation and infrared laser fixation were set as mentioned in the section above. The composite coatings were irradiated at corresponding power for 10 min and the temperature was recorded every 30 s. After 10 min, the laser was removed and the temperature was recorded every 30 s for another 10 min; finally, the heating and cooling temperature curve was drafted. The photothermal conversion efficiency refers to the reported literature [37]. The specific equation was as follows:

*η* = *hA*(Δ*T_max,mix_* − Δ*T_max,H_*_2__*O*_)/(*I*(1−10-Aλ)) × 100% (1)

*t* = −*md* × *Cd* × ln(θ)/*hA* (2)

θ = Δ*T*/*ΔT_mix,max_* (3)

η: photothermal conversion efficiency;
*h*: heat transfer coefficient;
*A*: surface area of the container; *t*: natural cooling time;
Δ*T_max, mix_*: maximum temperature difference between initial and 10 min;
${\Delta T}_{max,H_{2}O}$: maximum temperature difference in the PBS solution between initial and 10 min;
*m_d_* and *C_d_*: mass and specific heat capacity of PBS (close to the water phase), respectively;
Δ*T*: difference between the initial temperature and the cooling temperature;
*I*: infrared laser power.

### 2.8. Photothermal Conversion Stability Test

The sample (*n* = 3) was placed in a glass cuvette and immersed in 1 mL PBS. The laser was set at a horizontal distance of 2–3 cm from the sample, and the power was adjusted to 1 W·cm^−2^. Irradiation was started and the temperature was recorded every 30 s for 10 min. Later, the laser was removed, and the temperature was detected for another 10 min every 30 s. A total of 20 min was set as a cycle and was repeated 5 times. The temperature of PBS before irradiation was set as the initial temperature.

### 2.9. Singlet Oxygen Detection

First, 3.6 g of DPBF (1,3-diphenylisobenzofuran) was dissolved in 100 mL absolute ethanol to prepare the DPBF solution. Next, the composite coatings were immersed in 1.5 mL DPBF solution, and incubated in the dark for 5 min to achieve adsorption/desorption equilibrium under the irradiation of the 808 nm laser. Then, the irradiated solution was analyzed at 410 nm by UV-Vis spectroscopy. Each group was replicated three times (*n* = 3).

### 2.10. Antibacterial Effect Evaluation of Composite Coatings

Representative strains, *E. coli* and *S. aureus*, were seeded on the different substrates, including Ti, PPy@Cu, Cu/PD, and PPy@Cu/PD composite coatings, by the plate coating culture method to investigate the antibacterial efficacy. Each group had 3 parallel samples for statistical analysis (*n* = 3). The activated bacteria were diluted to 0.5 McF using saline, and 50 μL of this solution was dropped on the surface of composite coatings. After culturing in an incubator at 37 °C for 24 h, 10 μL of the bacterial solution was pipetted and diluted 200 times using saline, and 50 μL of the diluted solution was taken out to overlap the plates. The plate was put in the 37 °C incubator for 24 h, followed by a professional photographer obtaining pictures for qualitative and quantitative analysis of bacteria growth following the previously reported Equation (4) [38]. The photothermal synergistic antibacterial properties of the materials were also explored under irradiation conditions by taking the non-irradiated PPy@Cu/PD composite coating as the control group and the irradiated PPy@Cu/PD composite coating as the experimental group (*n* = 3). *E. coli* and *S. aureus* were seeded in MH medium and purified 3 times to harvest relatively pure colonies, respectively. And the PPy@Cu/PD composite coating was sterilized under UV light for 1 h and then fixed in a Petri dish. Single colonies of *E. coli* and *S. aureus* were scraped using an inoculating loop to prepare 0.5 McF bacterial suspensions with saline. An amount of 50 μL of *E. coli* and *S. aureus* bacterial suspensions was dropped on the surface of the PPy@Cu/PD composite coating, respectively, and irradiated with/without an 808 nm NIR laser at 1 W·cm^−2^. After 10 min, the Petri dishes were incubated at 37 °C for 6 h. Later, 10 μL of bacterial liquid was pipetted on PPy@Cu/PD with/without NIR irradiation and diluted 200 times using saline, of which 50 μL was pipetted to spread on plates, and then incubated at 37 °C for 24 h. Finally, the culture plates were taken out to take pictures, qualitative antibacterial analysis was conducted according to the proliferation of *E. coli* and *S. aureus* on the plates, and the number of colonies was counted to characterize the average bacteriostatic activity following the equation below:

R: bacteriostatic activity; *CCCG* and *CCEG*: colony count of the control group and treated group, respectively. 

### 2.11. In Vitro Effect of the Composite Coatings on Osteoblasts

OBs (Osteoblasts) were harvested from the calvaria of neonatal SD (Sprague Dawley) rats (2–3 days old). The animal method was approved by the Animal Care and Use Committee of the First Affiliated Hospital of Xinjiang Medical University (IACUC-20170222054). All protocols complied with the NIH (National Institutes of Health) *Guide for the Care and Utilization of Laboratory Animals*. Five groups with 7 parallel samples were set, including, Ti, PPy@Cu, Cu/PD, and PPy@Cu/PD. 

Using third-generation cells (Figure S1), titanium plates were placed in a 96-well cell culture plate. Osteoblasts were seeded onto a 1 cm × 1 cm titanium plate at a density of 3 × 10^4^ cells/mL, and 1 mL of cell suspension was added to each well to immerse the titanium plates. The plates were incubated at 37 °C in a 5% CO_2_ incubator for 1, 3, 5, and 7 days, and the medium was changed every 3 days. After the cells were incubated at the corresponding time point, the culture medium was discarded and washed with PBS three times. After removing the unattached cells, 4% paraformaldehyde solution was added to immerse the surface of the titanium plates and placed in a refrigerator at 4 °C for 1 h. The fixed solution was discarded and washed with PBS for 3 h. Next, the solution was dehydrated in gradient ethanol (30%, 50%, 75%, 90%, 100%) at room temperature for 10 min each, and finally dehydrated twice with anhydrous ethanol for 10 min each. After dehydration, the cells were naturally dried at room temperature in the shade. After CO_2_ critical point drying, they were treated with gold spray for 120 s to observe cell morphology and CCK-8.

Cells were electrically stimulated using a homemade high-throughput apparatus. The apparatus was a 24-well plate with a lid that was connected to the circuit with copper wires. Each hole on the plate had two copper wires that acted as electrodes. After plugging in an external power supply, the cells on the membrane were voltage-stimulated through these two copper wire electrodes (Table S2).

### 2.12. Statistical Analysis

Data are presented as mean ± standard deviation. Statistical analysis was carried out utilizing a one-sample *t*-test (assuming unequal variances). Differences (*p* < 0.05) between the two groups were considered significant.

## 3. Results and Discussion

### 3.1. Characterization of PPy@Cu/PD Composite Coatings

**Figure 1:**
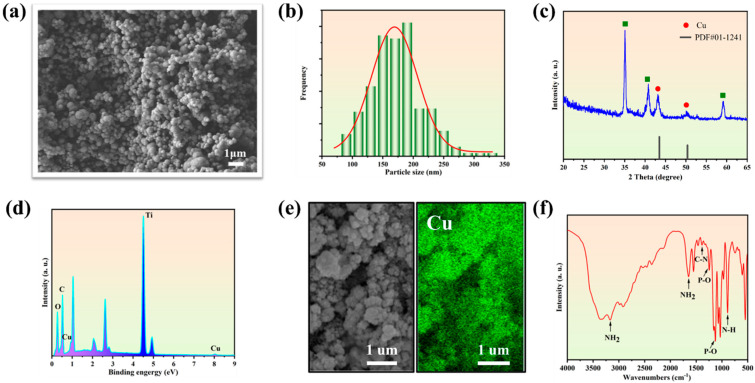
Characterization of PPy@Cu/PD. (**a**) The SEM image. (**b**) The diagram of particle size distribution. (**c**) XRD spectrum. (**d**) EDS energy spectrum analysis. (**e**) Surface scanning image. (**f**) FTIR spectrogram.

The PPy@Cu/PD composite coating showed spherical morphology (Figure 1a) with uniform nano-scale size ranging from 150 to 200 nm (Figure 1b). This is because, in the process of electrochemical deposition, Py lost electrons and was endowed with positive charges, generating Py radicals. The radicals were polymerized through the α and β positions and formed a positively charged PPy macromolecule [36]. Cu^2+^ was coordinated with the pyrrolic-N sites in the PPy macromolecule. When the voltage reached −1.5 V, the coordinated Cu^2+^ harvested electrons to generate Cu-NPs in situ so that the Cu-NPs were uniformly dispersed on the Ti substrate surface (cathode). The results of the XRD analysis (Figure 1c) revealed that the Cu diffraction peak that appeared in the composite coating was consistent with the PDF#01-1241 card. And the EDS analysis confirmed the presence of Cu and its uniform distribution (Figure 1d). To verify this, the mapping of the sample shows (Figure 1e) that the Cu element is evenly distributed on the coating surfaces. The FTIR spectrum (Figure 1f) shows the absorption peaks at 1292 cm^−1^ and 770 cm^−1^, which are generated by the C-N stretching vibration and the N-H ring out-of-plane bending in PPy [39,40]. The absorption peaks at 1068 cm^−1^, 1252 cm^−1^, 1645 cm^−1,^ and 3166 cm^−1^ represent the P-O stretching vibration, P=O stretching vibration, and NH_2_ bending and stretching vibration in the PD drug, respectively [27]. The results indicate that PPy@Cu /PD nanocomposite coatings were successfully prepared on the titanium surface using the PED method.

R = (*CCCG* − *CCEG*)/*CCCG* × 100% (4)

### 3.2. Electrochemical Measurement

Metal implants can be eroded to cause the release of metal ions, which come into contact with tissue and body fluids, thus reducing their performance. The efficiency and performance of metal implants are greatly influenced by their corrosion resistance properties. As shown in Figure S2, the corrosion behavior of composite coatings was evaluated by the Tafel curve. E_corr_ and I_corr_ were interpolated by the classical Tafel extrapolation (Table 1), and it can be observed that the PPy@/Cu/PD displayed better corrosion resistance than Ti, since the PPy@/Cu/PD exhibited the highest E_corr_ value and lowest I_corr_ value. It was reported that a Cu coating could decelerate the corrosion rate of metals [41]. Hence, all the above results proved that the PPy@/Cu/PD coating significantly inhibited the diffusion of the corrosion medium, thereby slowing the corrosion rate of the Ti substrate. 

### 3.3. Analysis of Ion Release and Antibacterial Properties of Composite Coatings

**Figure 2:**
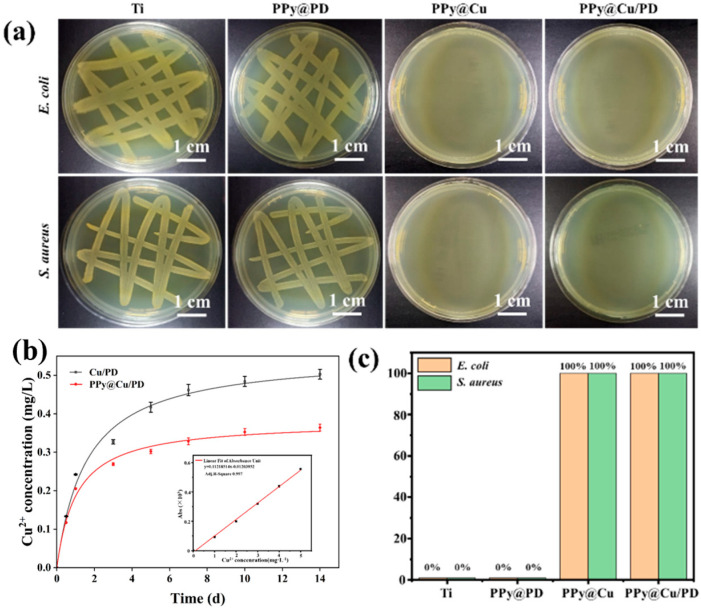
Assessment of the physiological stability and antibacterial properties of the composite coating. (**a**) Qualitative analysis of *E. coli* and *S. aureus*. (**b**) The release kinetic curve of Cu^2+^ (*n* = 3). (**c**) Qualitative analysis of *E. coli* and *S. aureus* antibacterial performance (*n* = 3).

As the qualitative analysis of bacteria proliferation shows in Figure 2a, after incubation for 24 h, the colonies of *E. coli* and *S. aureus* on Ti accumulated to form strains, while the bacterial colonies on the Cu-NP surfaces were significantly reduced. Moreover, the *E. coli* and *S. aureus* colonies were diminished on the PPy@Cu and PPy@Cu/PD composite coatings due to the introduction of PPy. The Cu^2+^ concentration is the essential factor of effective antibacterial action in the PPy@Cu/PD coating. The release profile of Cu^2+^ in the composite coating was investigated. It can be seen from Figure 2b that the release concentration of Cu^2+^ in the PPy@Cu/PD was significantly lower than that in the Cu/PD, and the amount of released Cu^2+^ decreased from 0.50 mg·L^−1^ to 0.36 mg·L^−1^. This was attributed to the modulatory effect of PPy on Cu-NPs, which achieved the stable and slow release of Cu^2+^. Figure S3 displays the release profile of PD^2−^. It was shown that the release rate of PD^2−^ in PPy@Cu/PD was lower than that in the Cu/PD composite coating. Incorporating PPy into the composite coating decreased the released PD^2−^ from 0.56 mg·L^−1^ to 0.39 mg·L^−1^. This is because, during the in situ oxidative polymerization of Py to PPy, the positive charges on the surface of Py attracted PD^2−^, which was incorporated into the PPy framework, achieving the stable and slow release of PD^2−^. The quantitative analysis in Figure 2c shows that the Ti with the PPy@PD coating has no antibacterial activity, with a 0% antibacterial activity. The antibacterial efficiency of the PD composite coating reaches 100%. Cu has superior antibacterial properties, which could destroy bacterial cell membranes, lipids, proteins, DNA, etc. [42]. The uniform dispersion of Cu-NPs by the regulation of PPy strengthened its antibacterial effect. These results proved that the PPy@Cu/PD composite coating had excellent physiological stability and antibacterial activity, enhancing the ability of Ti-based materials to prevent bone infection.

### 3.4. Analysis of Antibacterial Properties of PPy@Cu/PD Excited by NIR Laser

**Figure 3:**
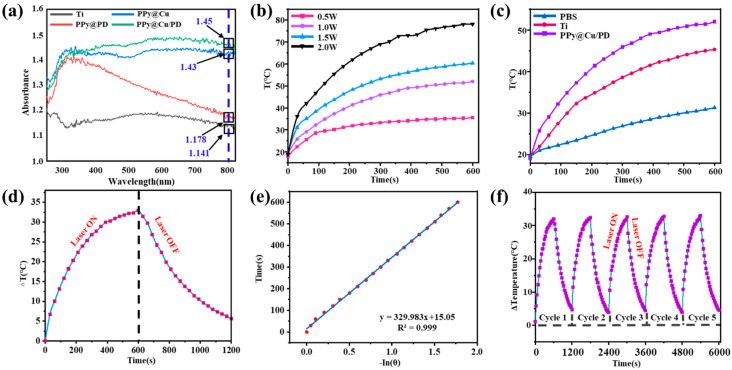
Analysis of photothermal properties of the composite coating. (**a**) UV-Vis absorption spectra of composite coatings. (**b**) Curves for temperature changes over time of PPy@Cu/PD composite coatings under different powers of 808 nm laser irradiation. (**c**) Variation curves of temperatures over time for different materials under 808 nm laser irradiation at a laser power of 1 W·cm^−2^. (**d**) Heating–cooling curves of PPy@Cu/PD composite coatings under 808 nm laser irradiation. (**e**) The relationship between cooling time and -ln(θ). (**f**) The cyclic heating curves of the composite coating under 808 laser irradiation at 1 W·cm^−2^ (*n* = 3).

To prevent bone infection in the clinic, it is necessary to shorten the antibacterial period and improve the surgical success rate. Cu-NPs exhibit strong light absorption in the NIR region owing to their plasmonic surface resonance efficacy and have excellent photothermal performance. Their temperature could rise to around 50 °C under NIR (808 nm) laser irradiation, which could kill bacteria by PTT without damaging normal tissues. In this work, the photothermal properties of the PPy@Cu/PD were characterized by being subjected to solid-state UV-vis-NIR in the wavelength ranging from 200 to 1000 nm. The results in Figure 3a show that the absorbance values of the PPy@PD coating, the PPy@Cu composite coating, the PPy@Cu/PD composite coating, and the Ti surface are 1.178, 1.43, 1.45, and 1.141, respectively. Compared with the Ti surface, the Cu NPs and PPy/Cu exhibit an SPR (surface plasmon resonance) effect in terms of absorption rate, and can absorb light of all wavelengths [43]. Therefore, the temperature-dependent curve of PPy@Cu/PD composite coatings under different powers of 808 nm NIR was explored. As shown in Figure 3b, the surface temperatures of the PPy@Cu/PD composite coatings were 35.5 °C, 52 °C, 60.4 °C, and 78.1 °C when irradiated with different powers. It has been reported that an NIR 808 nm laser could obliterate biofilms and eliminate bacteria both in vitro/vivo at high temperatures close to 50 °C without harming normal cells and tissues [44]. Thus, irradiation at 1 W·cm^−2^ was chosen as the preferred condition since the temperature reached under this power can achieve photothermal sterilization without causing significant damage to normal human tissues. The PBS solution, Ti, and PPy@Cu/PD composite coating were irradiated for 10 min at 1 W·cm^−2^ to investigate their temperature changes. As shown in Figure 3c, the temperatures of the PBS solution, the Ti, and the PPy@Cu/PD composite coating increased from room temperature to 31.3 °C, 45.3 °C, and 52 °C, respectively; the PPy@Cu/PD groups are significantly increased because of Cu-NPs, indicating that the PPy@Cu/PD was the fastest to reach the highest temperature that the human body can withstand. A temperature rising and falling test on the PPy@Cu/PD was carried out (Figure 3d). The cooling process was fitted and calculated referring to the photothermal conversion efficiency Equation (1), with the calculated value of 28% (Figure 3e). The cyclic photothermal performance of the composite coating was tested, and the results showed that its photothermal conversion capacity remained stable within five heating–cooling cycles (Figure 3f), indicating that the composite coating has excellent photothermal conversion stability.

**Figure 4:**
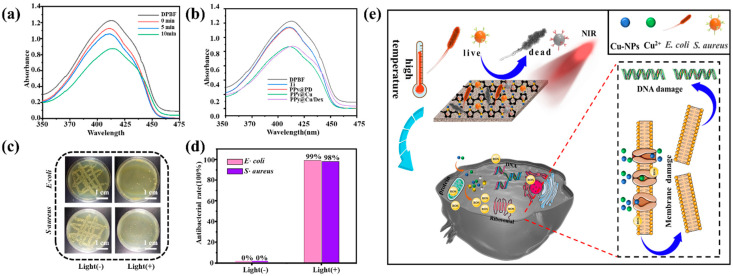
Analysis of the photothermal properties of the composite coatings. (**a**) The changes in ^1^O_2_ generated by the composite coatings under 808 nm irradiation at 1 W·cm^−2^ at different time points. (**b**) Variation in ^1^O_2_ in different composite coating groups at 1 W·cm^−2^ under 808 nm laser irradiation for 10 min. (**c**) Qualitative antibacterial images of PPy@Cu/PD surfaces cultured with bacteria for 6 h with/without irradiation. (**d**) Quantitative analysis of the antibacterial effect of PPy@Cu/PD composite coating cultured with bacteria for 6 h with/without irradiation. (**e**) Antibacterial mechanisms scheme (*n* = 3).

The ROS generated by Cu under NIR irradiation has broad-spectrum antibacterial activities and can penetrate bacteria walls and membranes to achieve antibacterial purposes. ^1^O_2_, a type of ROS, can react with DPBF to reduce its absorption strength at 410 nm. This reaction could be used to prove the existence of ^1^O_2_. As shown in Figure 4a, the absorbance gradually decreased over the illumination time. ROS production in different composite coatings was detected when exposed to light for 10 min (Figure 4b). The results exhibited that the absorbance of PPy@Cu/PD at 410 nm significantly decreased, indicating the generation of significant ROS. This is due to the SPR effect of Cu-NPs, which can produce local electron holes to generate ROS from H_2_O and O_2_ on the surface of the PPy@Cu/PD [45].

The effect of photothermal stimulation on the antibacterial properties was further evaluated. After incubating bacteria on the coating surface for 6 h, the coating surface was treated with a 1 W·cm^−2^ laser for 10 min to investigate the effect of photothermal stimulation on antibacterial effect. The qualitative antibacterial results in Figure 4c showed that when the PPy@Cu/PD composite coating was not irradiated, bacterial colonies were piled up, displaying the poorest antibacterial activities. However, no *E. coli* and *S. aureus* colonies were observed on PPy@Cu/PD after laser irradiation. Quantitative analysis (Figure 4d) further proved that the PPy@Cu/PD composite coating could inhibit the growth of *E. coli* and *S. aureus* by 99% and 98% only after 10 min of NIR irradiation. The temperature of the PPy@Cu/PD composite coating elevated after irradiation and reached the sterilization temperature of 52 °C, which could destroy bacterial metabolic signals, solidify their protein structures, change the function of enzymes, and affect the permeability of bacteria membranes, resulting in intracellular substances leakage and irreversible bacteria damage [46]. On the other hand, Cu-NPs have excellent bactericidal activity and can generate ^1^O_2_ under irradiation [32], which further enhances the photothermal antibacterial activity of the PPy@Cu/PD. The above synergistic effect can ensure the rapid and robust sterilization of this composite coating (Figure 4e).

### 3.5. Cytocompatibility of Composite Coatings

**Figure 5:**
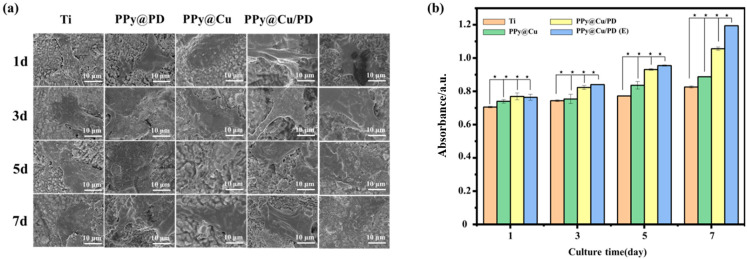
(**a**) SEM images of osteoblasts; (**b**) CCK−8 analysis of osteoblasts (*n* = 3). (^★^ indicates *p* < 0.05).

Osteoblasts are fundamental units in bone formation, so it is necessary to investigate the compatibility of the PPy@Cu/PD coating with osteoblasts. When the cells were cultured on the PPy@Cu/PD coating surface, the osteoblasts began to adhere on day 1, pseudopodia protruded from cell edges on day 3, and then spread well and sufficiently on day 5. The cells were connected and completed stretched out on the PPy@Cu/PD coating surface on day 7 (Figure 5a). 

To further boost the osteoinductive ability of the PPy@Cu/PD composite coating, a high-throughput device was introduced to electrically stimulate the osteoblasts. Compared with cells cultured in different groups from day 1 to day 7, it was found that with the increase in culture days, the non-electrically stimulated cells started floating up, and the spreading was insufficient (Figure 5a). In contrast, the electrically stimulated cells were fully spread and completely adhered to the coating material. It can be seen from the CCK−8 assay that from days 1 to 5, the cell viability on the coating with or without electrical stimulation showed no significant differences (Figure 5b). However, the cell activity in the electrically stimulated PPy@Cu/PD group was remarkably higher than that without electrical stimulation on day 7. This is due to the superior electrical responsiveness brought by the conductive polymer PPy. After electrical stimulation, the PPy-containing composite coating generates a weak electric field, which can activate the membrane system of the intracellular calcium store to promote the release of Ca^2+^. This could also increase the permeability of cell membranes to allow the influx of extracellular Ca^2+^ and increase the concentration of free Ca^2+^ in the cell fluid [47], thereby enabling the up-regulation of Ca^2+^ and activating the proliferation of osteoblasts. Overall, the osteoblast metabolic activity in the composite coating increased as the culture time was prolonged, and the addition of electrical stimulation endowed the PPy@Cu/PD composite coating with enhanced cytocompatibility.

## 4. Conclusions

In this study, a pulse electrochemical deposition technique was used to successfully construct a PPy@Cu/PD composite coating on a Ti surface with NIR photothermal responsive properties, achieving potent and durable antibacterial effects and promoting bone regeneration. The nano-sized Cu-NPs on the surfaces of the PPy@Cu/PD coating were uniformly dispersed. The release rate of Cu^2+^ from the composite coating was decreased with the regulation of PPy and the physiological stability was improved. This provides assurance for the long-lasting antibacterial capability for tissue replacement. Moreover, the PPy@Cu/PD composite coating exhibited an excellent photothermal effect with 28% photothermal conversion efficiency, as well as a high photothermal conversion stability in five heating–cooling cycles. The results of antibacterial assays proved that the bacteriostatic activity of the PPy@Cu/PD coating reached 100% after incubating with bacteria for 24 h. The photothermal synergistic antibacterial experiment demonstrated that the bacteriostatic activity of the PPy@Cu/PD coatings against *E. coli* and *S. aureus* reached 99% and 98% after 10 min of NIR irradiation, indicating that the rapid sterilization of the composite coating can be enhanced. The PPy@Cu/PD coating promotes the proliferation and differentiation of osteoblasts while maintaining excellent cell compatibility. In summary, the PPy@Cu/PD composite coating as a novel biomaterial realizes the potent and durable antibacterial and bone-inducing abilities of composite coatings. These should have a significant impact on applications in the field of tissue replacement.

## Figures and Tables

**Table 1:** E_corr_ and I_corr_ values of Ti and PPy@/Cu/PD.

	E_corr_ (V)	I_corr_ (A/cm^2^)
Ti	−0.78	1.584 × 10^−7^
PPy@/Cu/PD	−0.06	3.98 × 10^−7^

## Data Availability

The original contributions presented in the study are included in the article/Supplementary Materials, further inquiries can be directed to the corresponding author.

## Supplementary Materials

The following supporting information can be downloaded at: https://www.mdpi.com/article/10.3390/ma17153882/s1, Figure S1: Identification of osteoblasts. (a) Staining diagram of osteoblasts; (b) Alkaline phosphatase staining diagram; Figure S2: The release profile of PD2^−^; Figure S3: The potentiodynamic polarization plots of different samples in Ringer’s solution; Table S1: PBS solution formulation; Table S2: Electrical stimulation parameters.
