# Supplementary material for: Photothermal Antibacterial and Osteoinductive Polypyrrole@Cu Implants for Biological Tissue Replacement
Source: Materials (Basel). 2024 Aug 5;17(15):3882. doi: 10.3390/ma17153882 (PMC11313605; doi:10.3390/ma17153882)
Supplement: Supplementary file 1 [file materials-17-03882-s001.zip › materials-3077371-supplementary.pdf]

Supporting Information

Developing a Novel Potent-durable Antibacterial and Osteoinductive polypyrrole@Cu Coating with Multiple Antibacterial Mechanisms for Biological Tissue Replacement

Tianyou Zhou ‡1, Zeyan Zhou ‡3, Yingbo Wang *2

1 College of Control Engineering, Xinjiang Institute of Engineering, 1350 Aidinghu Road, Urumqi, Xinjiang, 830023, P. R. China; 40434196@qq.com

2 College of Chemical Engineering, Xinjiang Normal University, 102 Xinyi Road, Urumqi, Xinjiang, 830054, P. R. China; zhouzeyan@hnu.edu.cn

3 College of Materials Science and Engineering, Hunan University, 2 South Lushan Road, Changsha, Hunan, 410082, P. R. China; ybwang20002575@163.com

* Yingbo Wang; ybwang20002575@163.com

‡ Authors contributed equally.

* Corresponding author: 40434196@qq.com (T. Zhou); zhouzeyan@hnu.edu.cn (Z. Zhou); ybwang20002575@163.com (Y. Wang)

Table S1 PBS solution formulation

	Na^+	Ca^{2+}	Cl^-	HPO_4^{2-}
concentrations ($mmol·L^{-1}$)	142.0	12.5	217.0	5.0

Table S2. Electrical stimulation parameters

Type of electrical stimulation	Voltage	Frequency	Time
Single Stimulus	600mV	120 times	1200s

We confirmed that the cells extracted by the tissue block method used in this study were osteoblasts by morphological, cell staining (alkaline phosphatase) studies (Figure 1). The specific data are as follows:

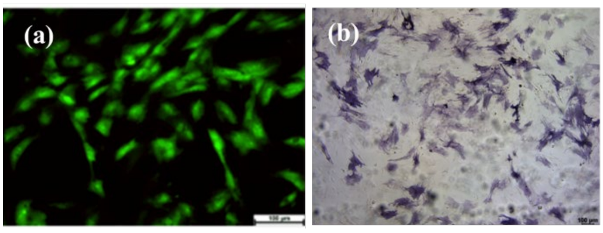

Fig. S1 Identification of osteoblasts. (a) Staining diagram of osteoblasts; (b) Alkaline phosphatase staining diagram.

Steps in the cell staining experiment:

(1.1) In vitro live and dead cell staining: Observe the cell morphology: 7 days after cell inoculation, discard the supernatant, wash with PBS 3 times, directly add the prepared Calcein AM/PI staining working solution, incubate for 30 minutes at room temperature and away from light, wash with PBS 3 times, and then observe the stained cells under the fluorescence microscope (green colour is for live cells).

(1.2) Alkaline phosphatase staining of osteoblasts: After the primary cells were passaged and grown for 3 generations, the 3rd generation osteoblasts were taken and

inoculated with 6-well plates at 5×10^4 wells, and when the cells grew to a suitable density, they were fixed with anhydrous ethanol for 5 min, rinsed with PBS for 3×5 min, and allowed to dry at room temperature. The cells were incubated at 37°C for 30 min after dropwise addition of freshly prepared matrix solution, and counted and analysed by microscopy.

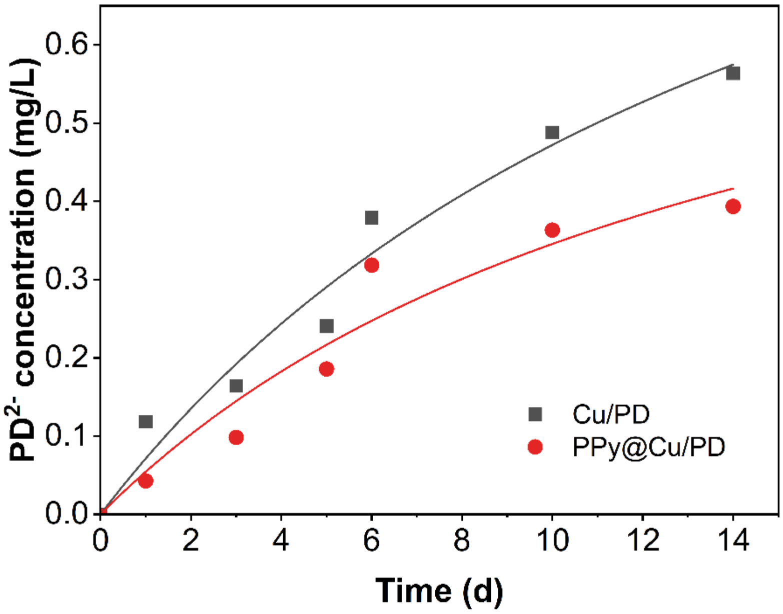

Fig S2. The release profile of PD^{2-}.

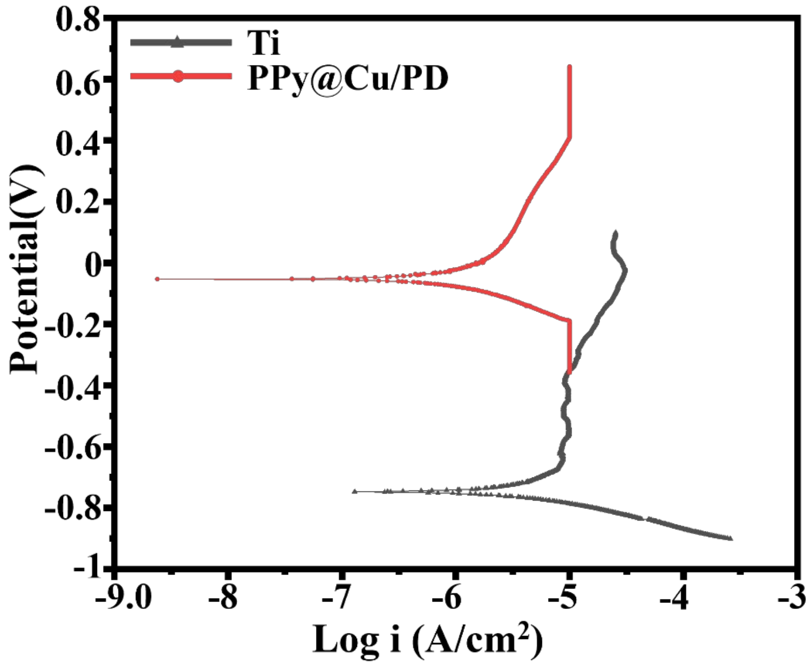

Fig S3. The potentiodynamic polarization plots of different samples in Ringer's solution.
